# Supplementary material for: Vulnerability of benthic trait diversity across the Mediterranean Sea following mass mortality events
Source: Nat Commun. 2025 Feb 12;16:1571. doi: 10.1038/s41467-025-55949-0 (PMC11821827; doi:10.1038/s41467-025-55949-0)
Supplement: Supplementary file 1 — Supplementary Information [file 41467_2025_55949_MOESM1_ESM.pdf]

Supplementary information of Carlot *et al.* (2025) Vulnerability of benthic trait diversity across the Mediterranean Sea following Mass Mortality Events

List of supplementary tables

Supplementary table 1 | Description of the different datasets used in this study.
Supplementary table 2 | Description of the information compiled from the T-MEDNet platform[1], Garrabou *et al.*, 2022[2] and the literature review.
Supplementary table 3 | Description of the ten traits used to measure trait diversity of benthic species.

List of supplementary figures

Supplementary figure 1 | PRISMA flow diagram of the systematic review process.
Supplementary figure 2 | Temporal distribution of Mass Mortality Events (MMEs) across the Mediterranean Sea.
Supplementary figure 3 | Distribution of trait categories within the 10 ecological traits studied for the 389 benthic species observed.
Supplementary figure 4 | Trait space occupied by Functional Entities (FEs) of benthic species across the Mediterranean Sea.
Supplementary figure 5 | Overview of the sampling effort and related measures.
Supplementary figure 6 | Trait space occupied by Functional Entities (FEs) of benthic species across three main regions (Western, Central, Eastern) of the Mediterranean Sea.

Data and code availability

All methods are described within the paper. The data to generate all figures is available at https://github.com/JayCrlt/MMEs_Mortality[3]

Supplementary table 1 | Description of the different datasets used in this study.

Dataset	Spanning range of the dataset	Taxon observed	Number of species	Number of observations	Reference used in this study
**Mass Mortality Event dataset**					
**T-MEDNet dataset**	2012-2020	Chlorophyta, Rhodophyta, Bryozoa, Porifera, Cnidaria, Mollusca, Echinodermata, Chordata	95	710	1
**Mortality dataset**	2015-2019	Ochrophyta, Rhodophyta, Tracheophyta, Bryozoa, Porifera, Cnidaria, Mollusca, Echinodermata, Chordata	70	1,125	2
**Systematic Review**	1986-2020	Chlorophyta, Ochrophyta, Rhodophyta, Tracheophyta, Bryozoa, Porifera, Cnidaria, Echinodermata, Chordata	58	266	this study
**Benthic Trait dataset**					
**Gomez-Gras *et al.*, 2021**	1999-2018	Rhodophyta, Bryozoa, Porifera, Cnidaria, Chordata	24		4
**Galobart *et al.*, 2023**	2011-2019	Chlorophyta, Ochrophyta, Rhodophyta, Tracheophyta	34		5
**Teixido *et al.*, 2024**	2018-2019	Chlorophyta, Ochrophyta, Rhodophyta, Tracheophyta, Bryozoa, Porifera, Cnidaria, Echinodermata, Chordata	196		6
**Golo *et al.*, 2024**	2000-2017	Chlorophyta, Ochrophyta, Rhodophyta, Bryozoa, Porifera, Cnidaria	62		7
**Current study**	1986-2020	Ochrophyta, Rhodophyta, Tracheophyta, Bryozoa, Porifera, Cnidaria, Mollusca, Echinodermata, Chordata	73		this study

Supplementary table 2 | Description of the information compiled from the T-MEDNet platform[1], Garrabou *et al.*, 2022[2] and the literature review. A total of 8 fields have been consistently identified and the corresponding examples and ranges are summarized in this table. See references at the end of the supplementary.

**Field**		**Field ranges and examples**	
**1)**	Scientific name	80 species (*e.g., Paramuricea clavata*, *Pinna nobilis*, *Cystoseira humilis*)	
	Phylum	9 phyla including Ochrophyta, Rhodophyta, Tracheophyta, Porifera, Cnidaria, Mollusca, Bryozoa, Echinodermata, and Chordata	
**2)**	Year	From 1986 to 2020	
**3)**	Longitude	From -5.4° to 35.0°	
	Latitude	From 31.4° to 45.6°	
**4)**	Depth range	From -180 m to 0 m	
**5)**	Type of habitat	5 types of habitats including hard substrates, seagrasses, mobile substrates, other type of substrates and non-available information	
**6)**	Mortality drivers	abiotic mortality drivers	**Temperature anomaly/Heat-related mortality**. They are usually defined as seawater temperature exceeding the 90th percentile threshold of the climatological mean for at least 5 consecutive days[2,8]
			**Storm**. Extreme rare and stochastic perturbations that can cause abrupt and dramatic ecological change within a short period of time[9]
			**Pollution**. It has been mainly characterized by oil spill from tankers affecting living species[10]
			**Increase of turbidity**. Human activities increase sedimentation as a result of coastal development such as dredging, land filling or runoff from coastal construction[11]
			**Other**. Other minor occasional mortality drivers in terms of frequency that we gathered (*e.g.,* fishing lines, flood)[12]
		biotic mortality drivers	**Diseases**. Frequent and severe disease outbreaks which are hypothesized to be a consequence of natural and anthropogenic stressors (*e.g.,* bacteria, protozoans)[13]
			**Mucilage cover**. Mucilaginous aggregates produced by planktonic or benthic algae considered as ecological threats[14]
			**Predator outbreak**. Due to human activities, outbreaks may result in important runaway consumption of resources[15]
			**Other**. Other minor occasional mortality drivers in terms of frequency that we gathered (*e.g.,* algal bloom)[16]
**7)**	Percentage of mortality	From 10% to 100%	
**8)**	Mortality category	Low impact (percentage of mortality below 30%)	
		Moderate impact (percentage of mortality between 30 and 60%)	
		Severe impact (percentage of mortality higher than 60%)	

Supplementary table 3 | Description of the ten traits used to measure trait diversity of benthic species. N indicates the number of categories within each trait. Genera and species in parenthesis are exhaustive examples of benthic organisms, but not necessarily from this study.

Trait	Trait Type	N	Categories
a) Feeding	Categorical	7	a) No, autotroph (*e.g.,* algae)<br>b) Active filter feeders with cilia (*e.g.,* bryozoans, sabellids)<br>c) Active filter feeders by pumping (*e.g.,* sponges, bivalves)<br>d) Passive filter feeders (*e.g.,* cnidarians)<br>e) Herbivores / Grazers (*e.g., Arbacia* sp., *Paracentrotus* sp.)<br>f) Carnivores (*e.g., Echinaster* sp., *Marthasterias* sp.)<br>g) Detritivores (*e.g., Holuthuria poli*)
b) Maximum longevity	Ordinal	5	1) < 1 - 1 year (*e.g., Dictyota* sp., *Halopteris scoparia*)<br>2) 2 - 5 years (*e.g., Cystoseira compressa*)<br>3) 5 - 10 years (*e.g., Ericaria balearica*, *Halimeda* sp.)<br>4) 10 - 20 years (*e.g., Pentapora* sp., *Paracentrotus* sp.)<br>5) ≥ 20 years (*e.g., Ericaria zosteroides*, Massive big sponges, *Paramuricea clavata, Corallium rubrum*)
c) Coloniality	Ordinal	2	1) Solitary (*e.g., Lithophyllum* sp., *Halocynthia* sp.)<br>2) Colonial/Modular/Gregarious (*e.g., Palmophyllum crassum,* Porifera, colonial corals, gorgonians, Bryozoa)
d) Morphological form	Categorical	12	a) Boring (*e.g., Cliona viridis*)<br>b) Encrusting, encrusting leaf-like (*e.g., Crambe crambe*)<br>c) Filamentous (*e.g., Ceramium* sp., *Cladophora* sp.)<br>d) Stolonial (*e.g., Caulerpa cylindracea*)<br>e) Foliose-erect (*e.g., Anadyomene stellata*)<br>f) Articulated (*e.g., Corallina* sp., *Jania* sp.)<br>g) Coarse branched (*e.g., Halopteris scoparia*)<br>h) Cup-like (*e.g., Leptopsammia* sp., *Caryophyllia* sp.)<br>i) Massive encrusting (height < radius; *e.g., Chondrosia* sp.)<br>j) Massive hemispheric (height = radius; *e.g., Ircinia oros*)<br>k) Massive erect (height > radius; *e.g., Halocynthia* sp.)<br>l) Tree-like (*e.g., Sargassum* sp., *Cystoseira* sp.)
e) Carbon storage	Categorical	3	a) Yes (*e.g., Posidonia oceanica*)<br>b) Potentially / Under discussion (*e.g., Cystoseira* sp.)<br>c) No (*e.g.,* macroinvertebrates, CCA)
f) Energetic resources	Ordinal	3	1) Photosynthetic autotroph (*e.g.,* algae)<br>2) Photo-heterotroph (*e.g., Cladocora caespitosa*)<br>3) Heterotroph (*e.g.,* most macroinvertebrates)
g) Size (Height)	Ordinal	5	1) Very small (< 2 cm) (*e.g., Palmophyllum* sp., *Cliona* sp.)<br>2) Small (2-5 cm) (*e.g., Plocamium* sp., *Mesophyllum* sp.)<br>3) Medium (5-20 cm) (*e.g., Halopteris* sp., *Padina* sp.)<br>4) Large (20-50 cm) (*e.g., Ericaria balearica*)<br>5) Very large (> 50 cm) (*e.g., Gongolaria montagnei*)
h) Growth rates	Ordinal	5	1) Extreme slow (< 1 $cm.yr^{-1}$) (*e.g., Corallium rubrum*)<br>2) Slow (*ca.* 1 $cm.yr^{-1}$) (*e.g., Cladocora* sp., *Paramuricea* sp.)<br>3) Moderate (> 1-5 $cm.yr^{-1}$) (*e.g., Myriapora* sp., *Astroides* sp.)<br>4) High (5-10 $cm.yr^{-1}$) (*e.g., Padina* sp., *Dictyota* sp.)<br>5) Very high (> 10 $cm.yr^{-1}$) (*e.g., Cladophora vagabunda*)
i) Calcification	Categorical	2	a) Without calcareous structure (*e.g., Cystoseira* sp., *Sargassum* sp., *Ulva* sp., demosponges)<br>b) With calcareous structures (*e.g., Padina* sp., *Halimeda* sp., calcareous sponges, gorgonians, corals)
j) Motility	Ordinal	2	1) Sessile (*e.g.,* algae and most macroinvertebrates)<br>2) Vagile (*e.g., Paracentrotus lividus*)

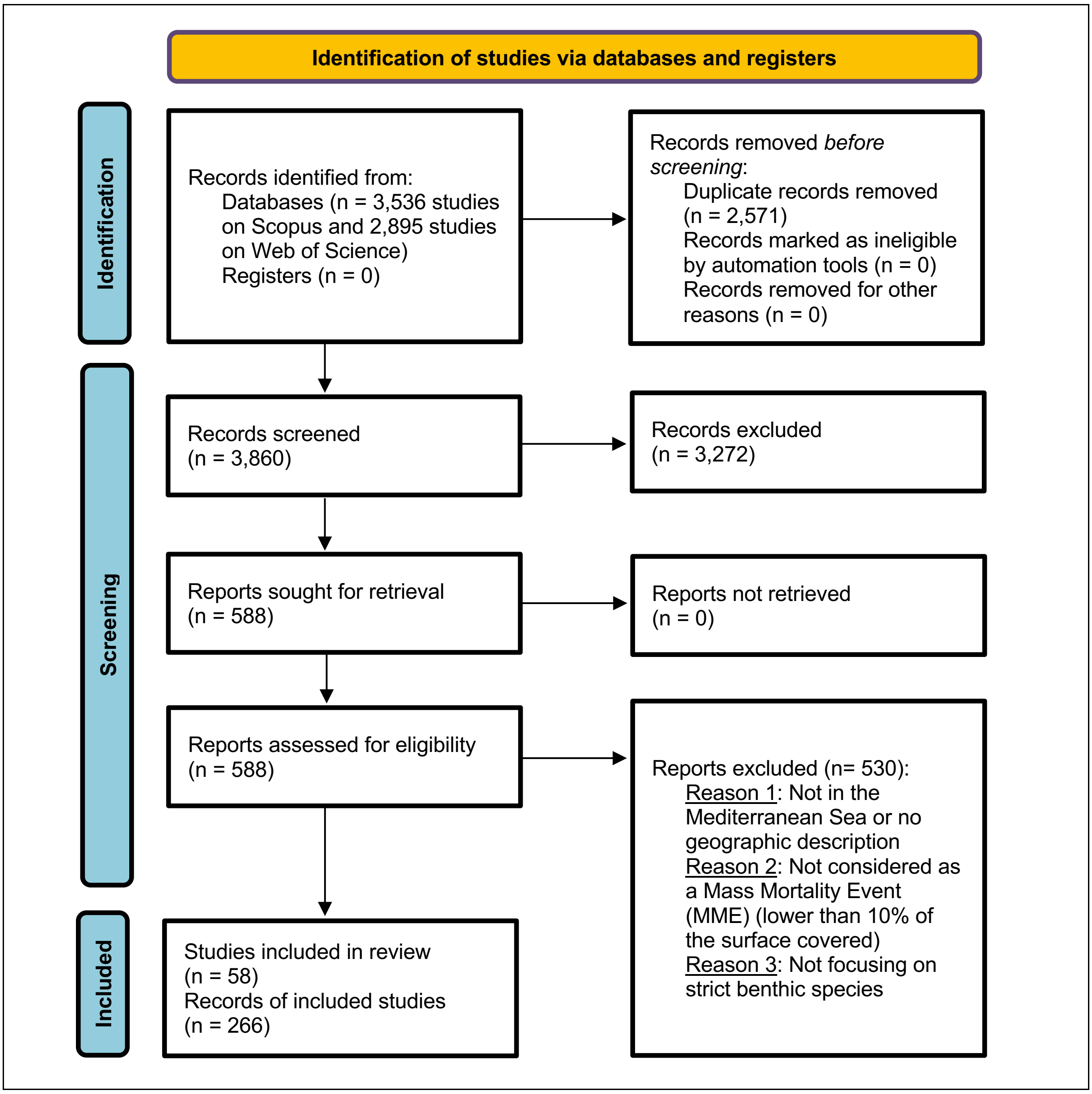

Supplementary figure 1 | PRISMA flow diagram of the systematic review process. The systematic review identified a total of 58 studies and 266 records, which were incorporated into a global dataset alongside the T-MEDNet platform[1] (n = 710 records) and Garrabou *et al.*, 2022[2] (n = 1,125 records) – see Supplementary Table 1. This combined global dataset includes 2,101 records of Mass Mortality Events (MMEs) in the Mediterranean Sea from 1986 to 2020. Of these, 243 records describe observed but unquantified MMEs, resulting in a final dataset of 1,858 records suitable for data analysis.

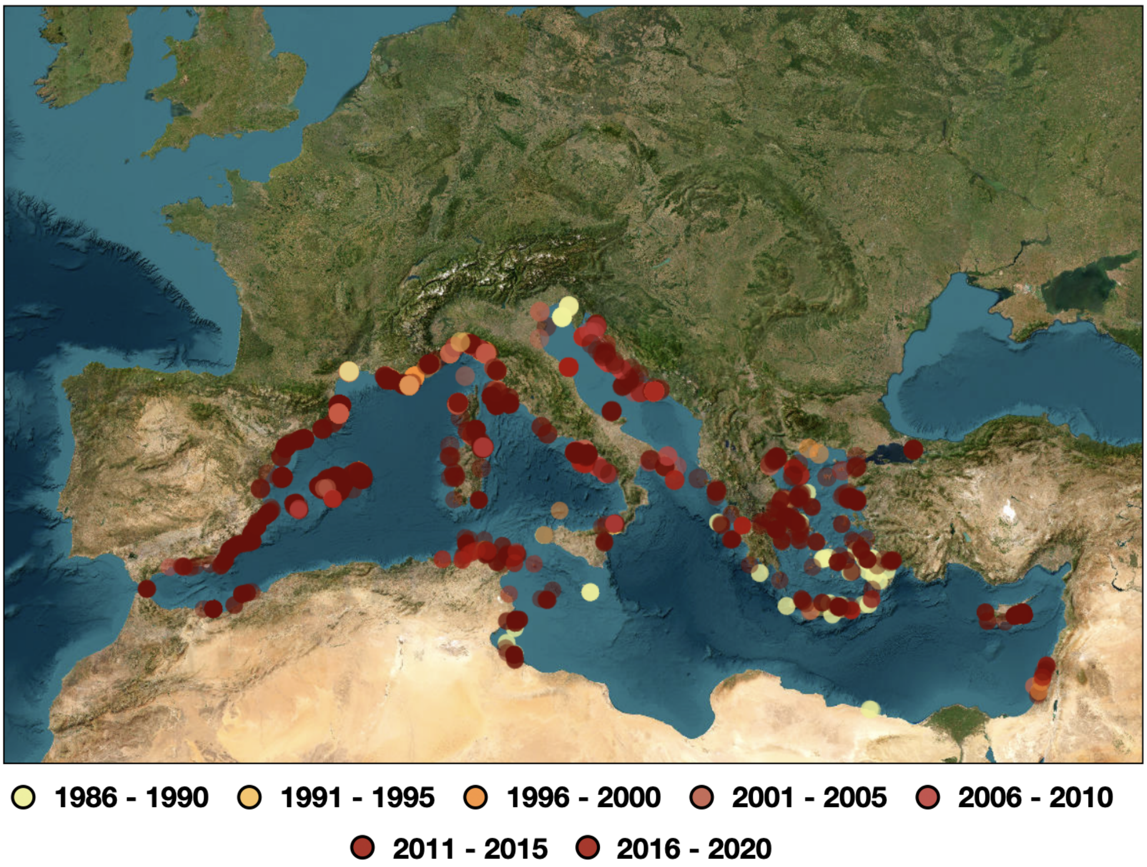

Supplementary figure 2 | Temporal distribution of Mass Mortality Events (MMEs) across the Mediterranean Sea. Spatial representation of 1,858 mortality records according to damage severity from 1986 to 2020. The color code is defined for a range of 5 years, with reddish colors representing the most recent MMEs observed and yellowish colors representing the older MMEs observed.

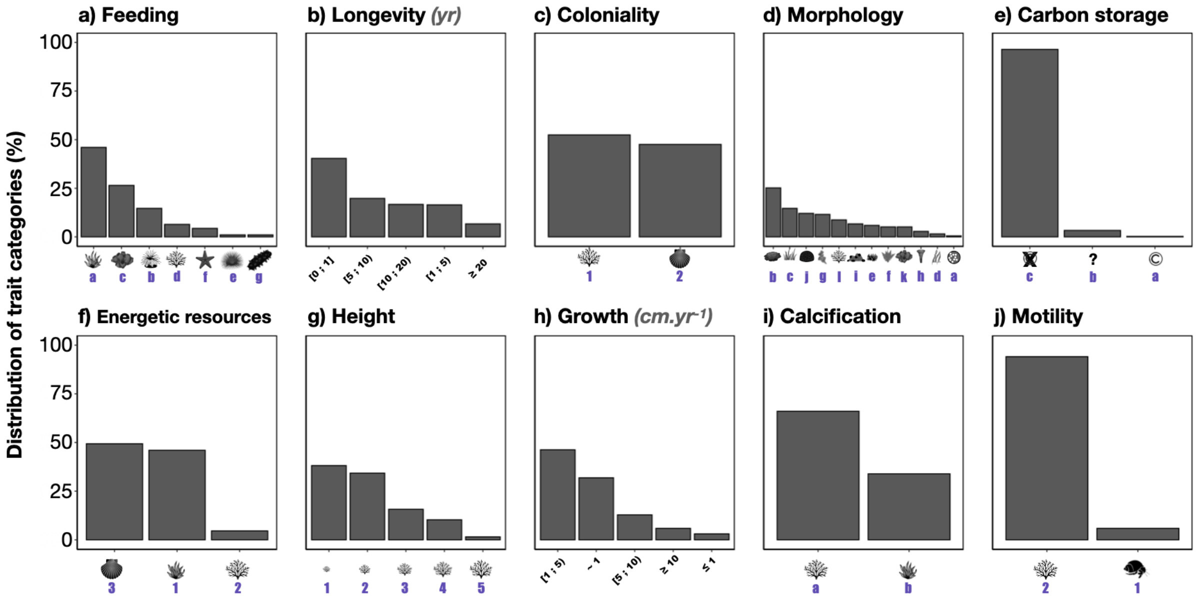

Supplementary figure 3 | Distribution of trait categories within the 10 ecological traits studied for the 389 benthic species observed. a. Feeding (a. no, autotrophs, b. active filter feeders with cilia, c. active filter feeders by pumping, d. passive filter feeders, e. herbivores and grazers, f. carnivores and g. detritivores), **b**, Maximum longevity ([0;1), [1;5); [5;10); [10;20); ≥ 20 years), **c**, Coloniality (1. colonial/modular, 2. solitary), **d**, Morphological form (b. encrusting, c. filamentous, f. articulated, h. cup-like, i. massive-encrusting, j. massive-hemispheric, k. massive-erect, l. tree-like), **e**, Carbon storage (a. yes, b. potentially, c. no), **f**, Energetic resources (1. photosynthetic autotroph, 2. photo-heterotroph, 3. heterotroph), **g**, Height (1. very small, 2. small, 3. medium, 4. large, 5. very large), **h**, Growth rate (≤1, ~1; [1;5); [5;10); ≥ 10 $cm.yr^{-1}$), **i**, Calcification (a. with calcareous structures, b. without calcareous structures), and **j**, Motility (1. sessile, 2. vagile). See Table S2 for trait category descriptions.

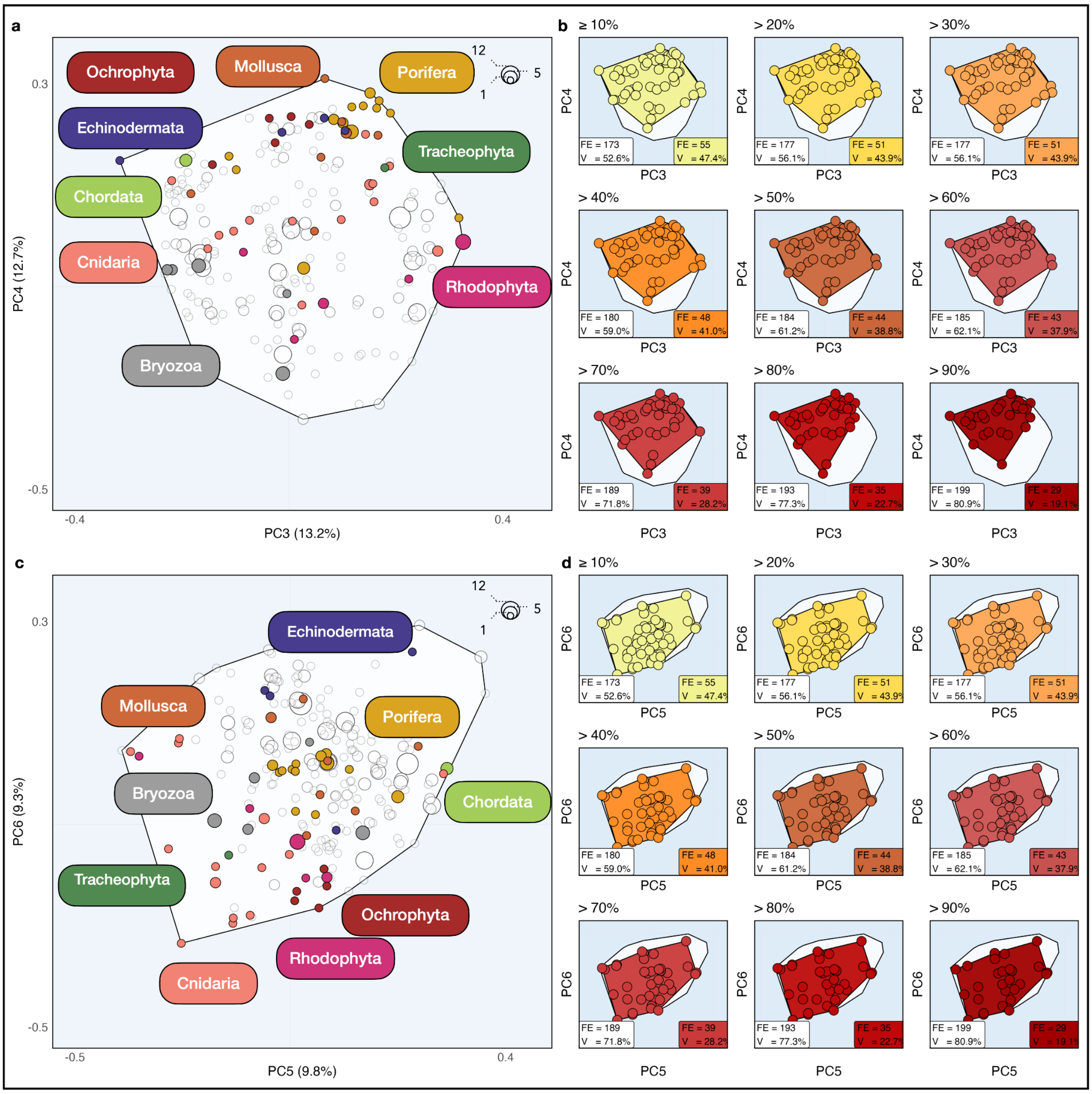

Supplementary figure 4 | Trait space occupied by Functional Entities (FEs) of benthic species across the Mediterranean Sea. The trait space for the global pool of 389 benthic impacted species and 228 FEs. Principal Coordinate Analysis (PCoA) was performed on trait values. **a, c.** Trait space of the global dataset according to PCoA 3 vs. 4. and PCoA 5 vs. PCoA 6, respectively. The size of the dots represents the number of species within a FE. **b, d**. Trait space following damage severity, which ranges from ≥ 10% to >90% damaged according to PCoA 3 *vs.* 4. and PCoA 5 *vs.* PCoA 6, respectively.

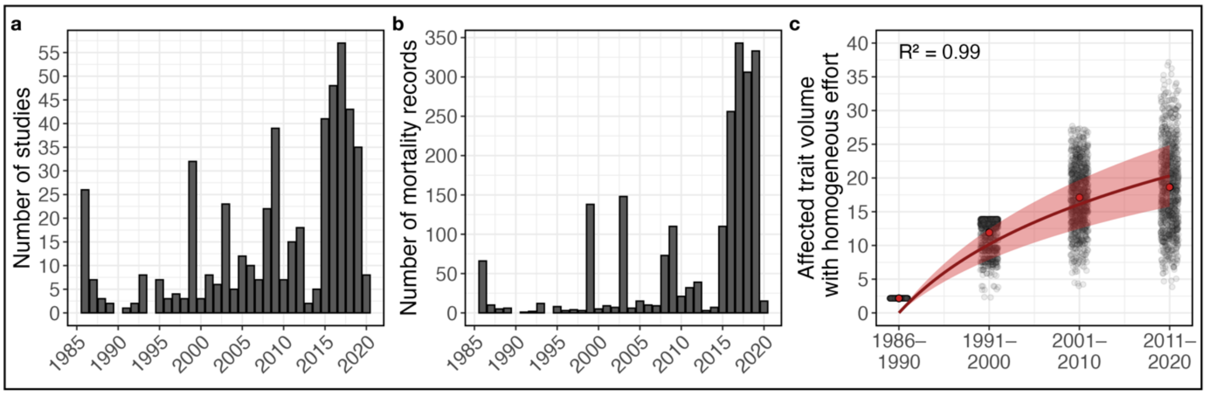

Supplementary figure 5 | Overview of the sampling effort and related measures. a. Number of studies assessing at least one MME over time, compiled in the Mass Mortality Event dataset. **b.** Number of mortality records assessed in the Mass Mortality Event dataset over time. **c.** Increase in the vulnerability of trait volume over time, based on random sampling of 100 mortality records per decade. This process has been repeated 1,000 times to strengthen our conclusions, with each iteration represented by a grey dot. The average value is indicated by a red dot. A logarithmic model fit is included to illustrate the increase in trait volume vulnerability over time, yielding an R^2 of 0.99.

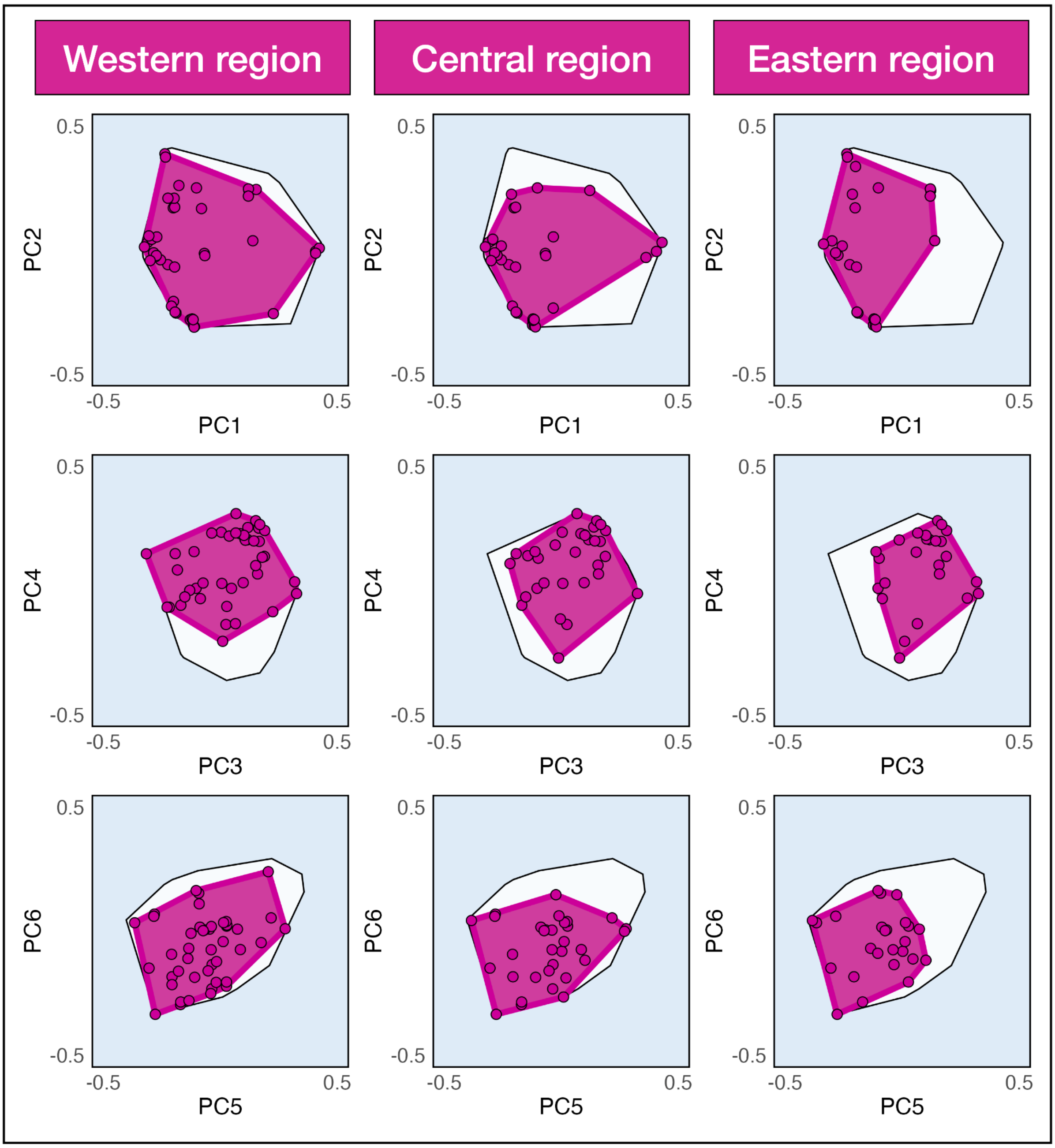

Supplementary figure 6 | Trait space occupied by Functional Entities (FEs) of benthic species across three main regions (Western, Central, Eastern) of the Mediterranean Sea. The trait space for the global pool of 389 benthic impacted species and 228 FEs. Principal Coordinate Analysis (PCoA) was performed on trait values.

References

1. Garrabou, J. *et al.* Collaborative Database to Track Mass Mortality Events in the Mediterranean Sea. *Front. Mar. Sci.* **6**, 707 (2019).
2. Garrabou, J. *et al.* Marine heatwaves drive recurrent mass mortalities in the Mediterranean Sea. *Glob. Change Biol.* **28**, 5708–5725 (2022).
3. Carlot, J. JayCrlt/MMEs_Mortality: Vulnerability of benthic trait diversity across the Mediterranean Sea following Mass Mortality Events. https://doi.org/10.5281/zenodo.14383070 (2024).
4. Gómez-Gras, D. *et al.* Climate change transforms the functional identity of Mediterranean coralligenous assemblages. *Ecol. Lett.* **24**, 1038–1051 (2021).
5. Galobart, C., Ballesteros, E., Golo, R. & Cebrian, E. Addressing marine restoration success: evidence of species and functional diversity recovery in a ten-year restored macroalgal forest. *Front. Mar. Sci.* **10**, 1176655 (2023).
6. Teixido, N. *et al.* Functional changes across marine habitats due to ocean acidification. *Glob. CHANGE Biol.* **30**, (2024).
7. Golo, R. *et al.* Decoding the enigma of Lophocladia: exploring its invasive behavior and the ecological implications. *Universitat de Girona* (2024).
8. Hobday, A. J. *et al.* A hierarchical approach to defining marine heatwaves. *Prog. Oceanogr.* **141**, 227–238 (2016).
9. Teixidó, N., Casas, E., Cebrián, E., Linares, C. & Garrabou, J. Impacts on Coralligenous Outcrop Biodiversity of a Dramatic Coastal Storm. *PLoS ONE* **8**, e53742 (2013).
10. Peirano, A., Damasso, V., Montefalcone, M., Morri, C. & Bianchi, C. N. Effects of climate, invasive species and anthropogenic impacts on the growth of the seagrass Posidonia oceanica (L.) Delile in Liguria (NW Mediterranean Sea). *Mar. Pollut. Bull.* **50**, 817–822 (2005).
11. Topçu, N. E. *et al.* Impact of excessive sedimentation caused by anthropogenic activities on benthic suspension feeders in the Sea of Marmara. *J. Mar. Biol. Assoc. U. K.* **99**, 1075–1086 (2019).
12. Piazzi, L., Cecchi, E., Cinti, M. F. & Ceccherelli, G. Extreme events and conservation of subtidal habitats: Effects of a rainfall flood on coralligenous reefs. *Mar. Pollut. Bull.* **165**, 112106 (2021).
13. Katsanevakis, S. The cryptogenic parasite Haplosporidium pinnae invades the Aegean Sea and causes the collapse of Pinna nobilis populations. *Aquat. Invasions* **14**, 150–164 (2019).
14. Piazzi, L. *et al.* Benthic mucilage blooms threaten coralligenous reefs. *Mar. Environ. Res.* **140**, 145–151 (2018).
15. Kružić, P., Lipej, L., Mavrič, B. & Rodić, P. Impact of bleaching on the coral Cladocora caespitosa in the eastern Adriatic Sea. *Mar. Ecol. Prog. Ser.* **509**, 193–202 (2014).
16. Sempere-Valverde, J. *et al.* Impacts of the non-indigenous seaweed Rugulopteryx okamurae on a Mediterranean coralligenous community (Strait of Gibraltar): The role of long-term monitoring. *Ecol. Indic.* **121**, 107135 (2021).
