# Supplementary material for: Vulnerability of benthic trait diversity across the Mediterranean Sea following mass mortality events
Source: Nat Commun. 2025 Feb 12;16:1571. doi: 10.1038/s41467-025-55949-0 (PMC11821827; doi:10.1038/s41467-025-55949-0)
Supplement: Supplementary file 3 — Description of Additional Supplementary Files [file 41467_2025_55949_MOESM3_ESM.pdf]

Description of Additional Supplementary Files of Carlot *et al.* (2025) Vulnerability of benthic trait diversity across the Mediterranean Sea following Mass Mortality Events

<u>**List of supplementary Data**</u>

Supplementary Data 1 | Bayesian Model Performance Overview. This table summarizes the output performance of our 11 models, presenting the overall accuracy for each, accompanied by error estimates and credible intervals at 2.5% and 97.5%. Additionally, detailed category-specific estimates, featuring values, error estimates, and credible intervals at the 2.5% and 97.5% confidence levels are provided. Model (11) Perturbations, represents the Bayesian general linear model, and showcases the estimates according to the average number of FEs impacted observed during the timeline. More information in Material and Methods.

Supplementary Data 2 | List of species affected with their corresponding trait categories. 389 species have been reported and characterized according to their feeding activity, maximum longevity, coloniality, morphological form, carbon storage, energetic resources, size, growth rates, calcification and motility. For each species, taxonomy has been reported. Sources are listed in the dataset column. More information on trait categories is provided in Supplementary table 2.
