# Supplementary material for: Vulnerability of benthic trait diversity across the Mediterranean Sea following mass mortality events
Source: Nat Commun. 2025 Feb 12;16:1571. doi: 10.1038/s41467-025-55949-0 (PMC11821827; doi:10.1038/s41467-025-55949-0)
Supplement: Supplementary file 6 — Reporting Summary [file 41467_2025_55949_MOESM6_ESM.pdf]

nature portfolio

Corresponding author(s): Jérémy Carlot

Last updated by author(s): Dec 13, 2024

Reporting Summary

Nature Portfolio wishes to improve the reproducibility of the work that we publish. This form provides structure for consistency and transparency in reporting. For further information on Nature Portfolio policies, see our Editorial Policies and the Editorial Policy Checklist.

Statistics

For all statistical analyses, confirm that the following items are present in the figure legend, table legend, main text, or Methods section.

n/a	Confirmed	
☐	☒	The exact sample size (*n*) for each experimental group/condition, given as a discrete number and unit of measurement
☒	☐	A statement on whether measurements were taken from distinct samples or whether the same sample was measured repeatedly
☒	☐	The statistical test(s) used AND whether they are one- or two-sided<br>*Only common tests should be described solely by name; describe more complex techniques in the Methods section.*
☒	☐	A description of all covariates tested
☒	☐	A description of any assumptions or corrections, such as tests of normality and adjustment for multiple comparisons
☐	☒	A full description of the statistical parameters including central tendency (e.g. means) or other basic estimates (e.g. regression coefficient) AND variation (e.g. standard deviation) or associated estimates of uncertainty (e.g. confidence intervals)
☒	☐	For null hypothesis testing, the test statistic (e.g. *F*, *t*, *r*) with confidence intervals, effect sizes, degrees of freedom and *P* value noted<br>*Give P values as exact values whenever suitable.*
☐	☒	For Bayesian analysis, information on the choice of priors and Markov chain Monte Carlo settings
☒	☐	For hierarchical and complex designs, identification of the appropriate level for tests and full reporting of outcomes
☒	☐	Estimates of effect sizes (e.g. Cohen's *d*, Pearson's *r*), indicating how they were calculated

Our web collection on statistics for biologists contains articles on many of the points above.

Software and code

Policy information about availability of computer code

Data collection	Data compiled from the systematic review is available online through GitHub (https://github.com/JayCrlt/MMEs_Mortality/tree/master/Data/R)
Data analysis	Code to reproduce analyses and figures is available online through GitHub (https://github.com/JayCrlt/MMEs_Mortality)

For manuscripts utilizing custom algorithms or software that are central to the research but not yet described in published literature, software must be made available to editors and reviewers. We strongly encourage code deposition in a community repository (e.g. GitHub). See the Nature Portfolio guidelines for submitting code & software for further information.

Data

Policy information about availability of data

All manuscripts must include a data availability statement. This statement should provide the following information, where applicable:

- Accession codes, unique identifiers, or web links for publicly available datasets
- A description of any restrictions on data availability
- For clinical datasets or third party data, please ensure that the statement adheres to our policy

All data and code to reproduce analyses and figures are available online through GitHub (https://github.com/JayCrlt/MMEs_Mortality/tree/master/Data/R)

Research involving human participants, their data, or biological material

Policy information about studies with human participants or human data. See also policy information about sex, gender (identity/presentation), and sexual orientation and race, ethnicity and racism.

Reporting on sex and gender	Not relevant
Reporting on race, ethnicity, or other socially relevant groupings	Not relevant
Population characteristics	Not relevant
Recruitment	Not relevant
Ethics oversight	Not relevant

Note that full information on the approval of the study protocol must also be provided in the manuscript.

Field-specific reporting

Please select the one below that is the best fit for your research. If you are not sure, read the appropriate sections before making your selection.

☐ Life sciences ☐ Behavioural & social sciences ☒ Ecological, evolutionary & environmental sciences

For a reference copy of the document with all sections, see nature.com/documents/nr-reporting-summary-flat.pdf

Ecological, evolutionary & environmental sciences study design

All studies must disclose on these points even when the disclosure is negative.

Study description	In this study, we have investigated the erosion of benthic trait diversity due to global and local mortality drivers, utilizing an unparalleled dataset of mass mortality events (MMEs) across the Mediterranean Sea, encompassing over 1,858 mortality records from 1986 to 2020. Leveraging several databases, we assembled the most comprehensive dataset of Mediterranean benthic species' traits, characterizing 389 species, based on 10 ecological traits, and resulting in 228 functional entities (FEs, i.e., species with the same combination of traits). Finally, we identified which FEs are the most affected and quantified the mortality drivers' contribution to their impairment over time and across regions.
Research sample	The research sample consists in mortality records observed in the Mediterannean sea from 1986 to 2020. In total, this study includes 1,858 mortality records across 747 unique locations of 73 benthic species, including 19 Porifera, 15 Cnidaria, 9 Rhodophyta, 9 Bryozoa, 8 Mollusca, 5 Ochrophyta, 4 Echinodermata, 3 Chordata, and 1 Tracheophyta.
Sampling strategy	Mortality data have been acquired from three main sources: (1) from the T-MEDnet platform since 2012 (https://t-mednet.org/massmortality/mass-mortality-events)51; (2) from Garrabou et al., (2022) from 2015 to 2019; and (3) a literature review carried out in November 2021 based on the Scopus and the Web of Science Core Collection databases
Data collection	Manuscripts were retrieved by applying the following research query: (mortalit* OR necros* OR diseas* OR bleaching OR heatwave* OR heat wave*) AND (marine OR sea OR ocean OR seabed OR benth* OR reef*) AND (Mediterranean OR Adriatic OR Levant* OR Aegean OR Alboran OR Tyrrhenian OR Balearic OR Ligurian OR Ionian OR Sicily OR Tunisian OR Sidra OR Catalan) to the title, keywords and abstract fields. As inclusion criteria only non-duplicated English manuscripts with empirical observations published between 1979 and 2020 were retained, leading to a total of 3,536 studies on Scopus and 2,895 studies on Web of Science to screen. We considered affected colonies or individuals displaying signs of recent mortality based on the following criteria: (1) denuded skeletons or tissue necrosis over 10% of its surface in gorgonians, sponges, and scleractinian corals and empty valves in bivalves attached to the substratum; and (2) increase of shoot mortality or sharp decline on thallus densities for seagrasses and habitat-forming macroalgal species. We compiled a total of 58 studies (n records = 264) leading to a dataset of 2,101 entries. Finally, for 243 entries, MMEs had been observed but not quantified, and were discarded, resulting in a final dataset of 1,858 entries.
Timing and spatial scale	From 1986 to 2020 across the entire Mediterranean Sea.
Data exclusions	We excluded all observations with a mortality rate lower than 10% and omitted all pelagic species. Additionally, any observations lacking quantifiable data were also excluded.
Reproducibility	This study does not consist in an experiment that can or not be reproduced. All the data and code needed to reproduce our study are made available.
Randomization	This study design does not consist in an experiment that requires randomization test. Our study is based on data on benthos traits that are used to estimate trait volume and quantify trait vulnerability
Blinding	This study design does not consist in an experiment that requires blinding during data acquisition. Our study is based on data on

benthos traits that are used to estimate trait volume and quantify trait vulnerability.

Did the study involve field work? ☐ Yes ☒ No

Reporting for specific materials, systems and methods

We require information from authors about some types of materials, experimental systems and methods used in many studies. Here, indicate whether each material, system or method listed is relevant to your study. If you are not sure if a list item applies to your research, read the appropriate section before selecting a response.

Materials & experimental systems

n/a	Involved in the study
☒	☐ Antibodies
☒	☐ Eukaryotic cell lines
☒	☐ Palaeontology and archaeology
☐	☒ Animals and other organisms
☒	☐ Clinical data
☒	☐ Dual use research of concern
☒	☐ Plants

Methods

n/a	Involved in the study
☒	☐ ChIP-seq
☒	☐ Flow cytometry
☒	☐ MRI-based neuroimaging

Animals and other research organisms

Policy information about studies involving animals; ARRIVE guidelines recommended for reporting animal research, and Sex and Gender in Research

Laboratory animals	This study did not involve laboratory animals.
Wild animals	This study involve wild animals but were not captured, only observed.
Reporting on sex	Not relevant
Field-collected samples	This study did not involve field-collected samples.
Ethics oversight	Not relevant

Note that full information on the approval of the study protocol must also be provided in the manuscript.

Plants

Seed stocks	Not relevant
Novel plant genotypes	Not relevant
Authentication	Not relevant
